# Neonatal anthropometric indicators of infant growth and mortality in Burkina Faso

**DOI:** 10.1017/S1368980024000880

**Published:** 2024-04-19

**Authors:** Mamadou Bountogo, Ali Sié, Alphonse Zakane, Guillaume Compaoré, Thierry Ouédraogo, Elodie Lebas, Kieran Sunanda O’Brien, Thomas M Lietman, Catherine E Oldenburg

**Affiliations:** 1 Centre de Recherche en Santé de Nouna, Nouna, Burkina Faso; 2 Francis I Proctor Foundation, San Francisco, CA, USA; 3 Department of Epidemiology & Biostatistics, University of California, San Francisco, CA, USA; 4 Department of Ophthalmology, University of California, San Francisco, CA, USA

**Keywords:** Undernutrition, Screening, Infant mortality, Underweight

## Abstract

**Objective:**

Most evidence supporting screening for undernutrition is for children aged 6–59 months. However, the highest risk of mortality and highest incidence of wasting occurs in the first 6 months of life. We evaluated relationships between neonatal anthropometric indicators, including birth weight, weight-for-age *Z*-score (WAZ), weight-for-length Z-score (WLZ), length-for-age *Z*-score (LAZ) and mid-upper arm circumference (MUAC) and mortality and growth at 6 months of age among infants in Burkina Faso.

**Design:**

Data arose from a randomised controlled trial evaluating neonatal azithromycin administration for the prevention of child mortality. We evaluated relationships between baseline anthropometric measures and mortality, wasting (WLZ < –2), stunting (LAZ < –2) and underweight (WAZ < –2) at 6 months of age were estimated using logistic regression models adjusted for the child’s age and sex.

**Setting:**

Five regions of Burkina Faso.

**Participants:**

Infants aged 8–27 d followed until 6 months of age.

**Results:**

Of 21 832 infants enrolled in the trial, 7·9 % were low birth weight (<2500 g), 13·3 % were wasted, 7·7 % were stunted and 7·4 % were underweight at enrolment. All anthropometric deficits were associated with mortality by 6 months of age, with WAZ the strongest predictor (WAZ < –2 to ≥ –3 at enrolment *v*. WAZ ≥ –2: adjusted OR, 3·91, 95 % CI, 2·21, 6·56). Low WAZ was also associated with wasting, stunting, and underweight at 6 months.

**Conclusions:**

Interventions for identifying infants at highest risk of mortality and growth failure should consider WAZ as part of their screening protocol.

Current WHO guidelines recommend the use of several indicators for identifying nutritionally at-risk in infants, including low weight-for-length *Z*-score (WLZ) and low weight-for-age *Z*-score (WAZ) in neonates^(1)^. Infants with low WLZ, or wasting (WLZ < –2), are at increased risk of morbidity and mortality compared with those with WLZ ≥ –2^(2)^. Recently, evidence has suggested that WAZ and mid-upper arm circumference (MUAC) may be the best predictors of morbidity and mortality in infants under 6 months of age^(3)^. In children over 6 months of age, acute malnutrition is defined based on WLZ (or weight-for-height *Z*-score, WHZ) or MUAC, and guidelines for management are based on moderate (WLZ < –2 or MUAC < 12·5 cm) and severe acute malnutrition (WLZ < –3 or MUAC < 11·5 cm)^(4)^. In children aged 6–59 months, both WAZ and MUAC are stronger predictors of mortality compared with WLZ/WHZ, and increasing attention has turned to the use of WAZ to identify nutritionally at-risk children^(5,6)^. Significantly less evidence exists to guide policy related to identification of at-risk infants under 6 months of age compared with children over 6 months of age.

Peak incidence of wasting in childhood occurs between birth and 3 months of age^(7)^. These infants struggle to catch up in growth trajectories to children who do not experience wasting, suggesting that an early wasting episode may disadvantage children for life, even if they do recover from wasting. Early identification of infants who are nutritionally at risk may allow for early intervention for these children.

The Sahelian region of West Africa is particularly vulnerable to undernutrition due to seasonal food insecurity, political instability and climate change that can shorten growing seasons^(8)^. We used data from a randomised controlled trial of neonatal azithromycin administration for the prevention of infant mortality in Burkina Faso^(9)^ that included anthropometric measurements at baseline and 6 months of age to evaluate the ability of neonatal anthropometric measurements and birth weight to predict mortality and undernutrition (including wasting, stunting and underweight) at 6 months of age.

## Methods

### Parent study

The *Nouveaux-nés et Azithromycine: une Innovation dans le Traitement des Enfants* (NAITRE) study was a 1:1 randomised controlled trial evaluating the efficacy of a single oral 20 mg/kg dose of azithromycin compared with placebo administered to neonates aged 8–27 d of age for prevention of all-cause infant mortality^(9,10)^. Infants were followed at 6 months of age to assess anthropometric outcomes and vital status.

### Study setting

Participants were enrolled in five regions of Burkina Faso, a landlocked country in the West African Sahel region. Burkina Faso experiences highly seasonal rainfall, with a rainy season from approximately June through October that coincides with the high malaria transmission season. Food insecurity is typically higher during this period, as the annual harvest occurs in approximately November^(11)^.

### Participants

Although azithromycin was hypothesised to reduce all-cause mortality in neonates based on results of trials in older infants^(12)^, some evidence from observational studies has suggested that exposure to macrolides during the neonatal period may increase the risk of infantile hypertrophic pyloric stenosis, a rare but serious condition that requires surgical intervention^(13)^. As a result, while the primary aim of the parent trial was to assess the efficacy of azithromycin for mortality, inclusion criteria focused on safety. Eligible participants were between 8 and 27 d of age and weighed at least 2500 g at the time of enrolment, as these subgroups of neonates were thought to have reduced risk of pyloric stenosis. Participants were enrolled in primary care facilities that were within 4 h of a tertiary care facility that had paediatric surgical capacity. Additional inclusion criteria included ability to feed orally, planning to remain in the study area for the duration of the study and caregiver consent.

### Anthropometric measurements

Birth weight measurements were extracted from each child’s government-issued health card. Birth weight is routinely recorded in the government health cards for all children who are delivered in facilities. Because birth weight was extracted from existing records, the measurements were not standardised or collected by trained study staff. Anthropometric measurements (height, weight and MUAC) were measured at enrolment (age 8–27 d) and at 6 months of age. Weight was measured using a standard infant scale. The scale was standardised each morning prior to measurement of any infants using a 2-kg test weight. Length was measured in triplicate using a ShorrBoard (Weight and Measure, LLC, Olney, MD), and the median was used for analysis. MUAC was measured using a standard MUAC tape (Weight and Measure, LLC, Olney, MD). WAZ, WLZ and length-for-age *Z*-score (LAZ) were calculated based on 2006 WHO growth standards^(14)^. We defined moderate (WLZ < –2 to ≥ –3) and severe (WLZ ≤ –3) wasting, moderate (LAZ < –3 to ≥ –3) and severe (LAZ < –3) stunting, and moderate (WAZ < –2 to ≥ –3) and severe (WAZ < –3) underweight at baseline to assess each measure’s predictive ability for predicting mortality and moderate wasting, stunting and underweight at 6 months of age. There are no currently accepted cut-offs for MUAC for infants under 6 months of age, and previous studies have found a range of cut-offs from 10·5 to 11·5 cm for identifying infants at highest risk of mortality. We therefore used cut-offs of ≥11·5 cm, <11·5 to ≥10·5 cm, <10·5 to ≥9·5 cm and <9·5 cm. Birth weight was categorised into low birth weight (<2500 g) or normal birth weight (≥2500 g).

### Vital status

Vital status was measured at the 6-month follow-up visit. Children were classified as died, alive or unknown. Vital status at 6 months of age was used as the main outcome for analysis.

### Statistical methods

We evaluated the relationship between birth weight (pre-enrolment), WAZ, WLZ, LAZ and MUAC (at enrolment, 8–27 d of age) and 6-month outcomes, including mortality, wasting, stunting and underweight. We built a separate logistic regression model for each outcome and baseline anthropometric measurement, adjusted for age at enrolment and sex of the infant, to assess the relationship between predefined categories of anthropometric measures at baseline and each outcome at 6 months. To assess the discrimination of each baseline anthropometric measure to identify mortality risk, we calculated the area under the receiving operating characteristic curve. Because the study treatment (azithromycin or placebo) occurred after baseline anthropometric measurements, and because there was no effect of azithromycin *v*. placebo on mortality or anthropometric endpoints, analyses were not adjusted for the study treatment arm^(9)^. All analyses were conducted in R version 4.1.3 (The R Foundation for Statistical Computing).

## Results

Of 21 832 infants enrolled in the trial, 7·9 % were low birth weight (weight < 2500 g) based on data extracted from their government-issued health card. Birth weight information was missing for 512 (2·4 %) infants. At enrolment (8–27 d of age), 7·4 % were underweight (WAZ < –2), 13·3 % were wasted (WLZ < –2) and 7·7 % were stunted (LAZ < –2; Table 1). Vital status information at 6 months of age was available for 20 960 children. By 6 months of age, ninety-two of the enrolled children had died (0·44 %). At 6 months of age, 7·0 % of infants were underweight, 5·8 % were wasted and 9·3 % were stunted.

**Table 1:** Baseline characteristics (*n* 21 832) of the study sample

	Characteristic	% or sd
Female sex, *n* (%)	10 844	49·7 %
Age at enrolment, days, median (IQR)	11	9, 14
Breast-feeding		
Immediate	20 661	94·6 %
Delayed	1140	5·2 %
Not breast-feeding	26	0·1 %
Missing	5	0·02 %
Birth weight, g		
Mean (s d)	2998	423
Birth weight < 2500 g	1719	7·9 %
Missing	512	2·4 %
WAZ at enrolment		
Mean (s d)	−0·61	0·94
WAZ < –2	1617	7·4 %
Missing	1	0·005 %
WLZ at enrolment		
Mean (s d)	−0·64	1·3
WLZ < –2	2903	13·3 %
Missing	56	0·3 %
LAZ at enrolment		
Mean (s d)	−0·52	1·06
LAZ < –2	1673	7·7 %
Missing	4	0·02 %
MUAC at enrolment, cm		
Mean (s d)	10·9	1·1
Missing	86	0·4 %

IQR, interquartile range; WAZ, weight-for-age *Z*-score; WLZ, weight-for-length *Z*-score; LAZ, length-for-age *Z*-score; MUAC, mid-upper arm circumference.

**Fig. 1:**
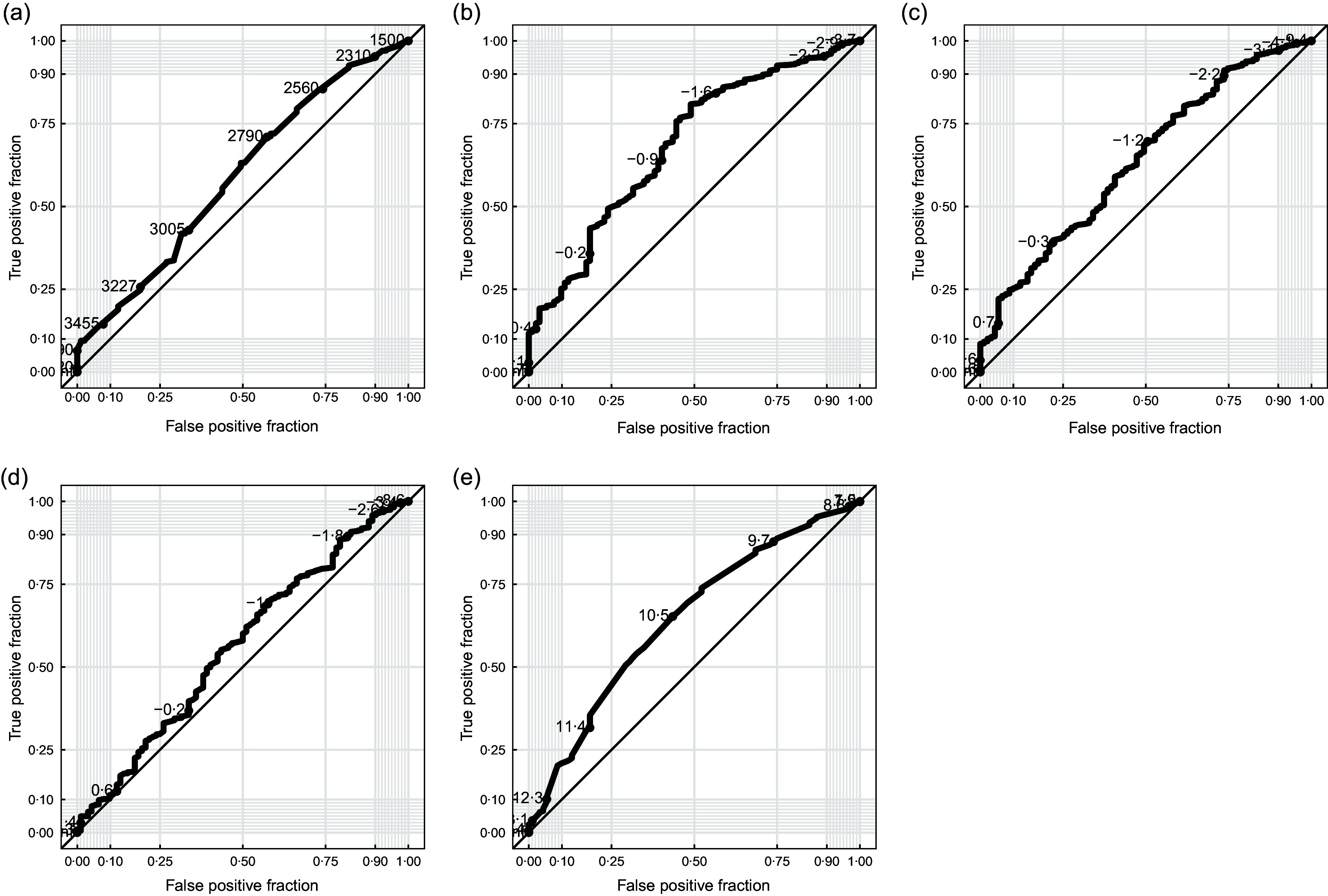
Receiving operator characteristic curves for neonatal anthropometric measurements predicting mortality by 6 months of age, including (a) birth weight, (b) weight-for-age *Z*-score, (c) weight-for-length *Z*-score, (d) height-for-age *Z*-score and (e) mid-upper arm circumference

All baseline anthropometric measurements were associated with mortality, with children with greater anthropometric deficits for all indicators at baseline having increased risk of mortality by 6 months of age (Table 2). The strongest predictor of mortality in all categories was WAZ, which also had the greatest area under the Receiver Operator Characteristic (ROC) curve (0·68, 95 % CI 0·63, 0·74; Table 2; Fig. 1). Predefined categories for MUAC as an indicator of undernutrition are not established for infants under 6 months of age. We found evidence of increasing risk of mortality as MUAC decreased. There was no evidence of effect modification by low birth weight, although the analysis was underpowered, especially for the low birth weight subgroup (see online supplementary material, Supplemental Table S1). In the overall cohort, a cut-off of 10·5 cm appeared to have the best performance for detecting children at high risk of mortality (Fig. 1).

**Table 2:** Associations between baseline anthropometric measures and mortality at 6 months

	*n*	Number died	%	OR*	95 % CI
Birth weight					
≥2500 g	18 807	73	0·37 %	1·00	
<2500 g	1654	16	0·93 %	2·57	1·42, 4·36
AUC (continuous)				0·56	0·50, 0·62
WAZ					
≥ –2	19 405	70	0·35 %	1·00	
< –2 and ≥ –3	1406	18	1·23 %	3·91	2·21, 6·56
< –3	148	4	2·55 %	11·15	2·97, 34·27
AUC (continuous)				0·68	0·63, 0·74
WLZ					
≥ –2	18 094	65	0·34 %	1·00	
< –2 and ≥ –3	2049	14	0·66 %	1·92	1·03, 3·32
< –3	762	12	1·51 %	4·44	2·28, 7·96
AUC (continuous)				0·64	0·58, 0·70
LAZ					
≥ –2	19 362	81	0·40 %	1·00	
< –2 and ≥ –3	1344	7	0·50 %	1·24	0·51, 2·54
< –3	250	4	1·53 %	3·82	1·13, 9·69
AUC (continuous)				0·56	0·50, 0·62
MUAC					
≥ 11·5 cm	6419	16	0·24 %	1·00	
< 11·5 to ≥ 10·5 cm	7177	24	0·32 %	1·38	0·73, 2·64
< 10·5 to ≥ 9·5 cm	5790	38	0·64 %	2·78	1·57, 5·16
< 9·5 cm	1489	14	0·91 %	3·96	1·90, 8·17
AUC (continuous)				0·61	0·55, 0·67

WAZ, weight-for-age *Z*-score; WLZ, weight-for-length *Z*-score; LAZ, length-for-age *Z*-score; MUAC, mid-upper arm circumference.

* Adjusted for child’s age in days at enrolment and sex.

All anthropometric deficits at baseline were associated with anthropometric deficits at 6 months of age, including wasting, stunting and underweight (Table 3). In general, children with specific anthropometric deficits at enrolment had higher risk of that deficit at 6 months of age (e.g. wasting). Low WAZ at enrolment and low birth weight were predictors of all anthropometric deficits at 6 months of age (Table 3). Low MUAC at enrolment was predictive of wasting and underweight at 6 months of age, but only MUAC < 9·5 cm was associated with stunting (Table 3).

**Table 3:** Associations between baseline anthropometric indicators and wasting, stunting and underweight at 6 months of age

	Wasted (WLZ < –2)				Stunted (LAZ < –2)				Underweight (WAZ < –2)			
	*n*	%	OR*	95 % CI	*n*	%	OR*	95 % CI	*n*	%	OR*	95 % CI
Birth weight												
≥2500 g	959	4·9 %	1·00		1450	7·4 %	1·00		1105	5·6 %	1·00	
<2500 g	123	7·2 %	1·49	1·22, 1·81	303	17·6 %	2·99	2·59, 3·45	212	12·3 %	2·35	1·99, 2·75
AUC (continuous)			0·56	0·54, 0·58			0·58	0·57, 0·60			0·60	0·58, 0·62
WAZ												
≥ –2	972	4·8 %	1·00		1477	7·3 %	1·00		1083	5·4 %	1·00	
< –2 and ≥ –3	129	8·8 %	1·88	1·54, 2·29	268	18·4 %	2·79	2·40, 3·24	231	15·8 %	3·11	2·65, 3·64
≤ –3	11	7·0 %	1·53	0·76, 2·79	43	27·4 %	5·15	3·43, 7·64	32	20·4 %	3·93	2·52, 5·98
AUC (continuous)			0·62	0·61, 0·64			0·64	0·63, 0·66			0·70	0·68, 0·71
WLZ												
≥ –2	815	4·3 %	1·00		1544	8·2 %	1·00		1069	5·7 %	1·00	
< –2 and ≥ –3	218	10·3 %	2·53	2·16, 2·96	169	8·0 %	0·99	0·83, 1·16	197	9·3 %	1·74	1·48, 2·04
≤ –3	79	10·0 %	2·56	1·99, 3·25	58	7·3 %	0·92	0·67, 1·20	75	9·5 %	1·83	1·41, 2·33
AUC (continuous)			0·65	0·63, 0·66			0·47	0·46, 0·49			0·58	0·56, 0·59
LAZ												
≥ –2	1023	5·1 %	1·00		1410	7·0 %	1·00		1121	5·6 %	1·00	
< –2 and ≥ –3	79	5·6 %	1·08	0·85, 1·37	294	20·8 %	3·70	3·19, 4·29	180	12·8 %	2·35	1·97, 2·79
≤ –3	10	3·8 %	0·71	0·35, 1·28	84	32·2 %	7·14	5·33, 9·52	45	17·2 %	3·16	2·22, 4·42
AUC (continuous)			0·50	0·48, 0·52			0·69	0·67, 0·70			0·65	0·63, 0·66
MUAC												
≥ 11·5 cm	229	3·4 %	1·00		597	8·9 %	1·00		272	4·1 %	1·00	
< 11·5 to ≥ 10·5 cm	426	5·7 %	1·76	1·50, 2·08	466	6·2 %	0·71	0·63, 0·81	461	6·1 %	1·67	1·43, 1·95
< 10·5 to ≥ 9·5 cm	346	5·8 %	1·84	1·55, 2·19	521	8·7 %	1·09	0·96, 1·23	433	7·2 %	2·13	1·82, 2·50
< 9·5 cm	107	7·0 %	2·22	1·75, 2·82	193	12·5 %	1·65	1·38, 1·97	172	11·2 %	3·41	2·78, 4·18
AUC (continuous)			0·53	0·52, 0·55			0·48	0·46, 0·49			0·55	0·54, 0·57

WAZ, weight-for-age *Z*-score; WLZ, weight-for-length *Z*-score; LAZ, length-for-age *Z*-score; MUAC, mid-upper arm circumference.

* Adjusted for child’s age in days at enrolment and sex.

## Discussion

Consistent with previous evidence, we found that WAZ was the strongest predictor of mortality among infants under 6 months of age in Burkina Faso^(3)^. Results are consistent with a previous study in Burkina Faso, which found that MUAC and WAZ at birth better identify risk of mortality than WLZ^(15)^. WAZ is attractive as an alternative anthropometric indicator for mortality compared with WLZ, as it does not require measuring length, which can be more error-prone than measuring weight. Previous evidence has shown lower reliability of WLZ compared with WAZ or MUAC measurements^(16)^. Weight is routinely measured in primary care settings for monitoring growth in infancy using standard hanging infant scales. Using these measures to identify children at highest risk of poor outcomes and engage them in care could leverage existing health infrastructure for screening children for undernutrition. However, although WAZ is typically easier to measure than WLZ, in practice, hanging infant scales may have low precision for measuring young infants, which can introduce bias into the measurement of WAZ.

For children aged 6–59 months, MUAC is routinely used in community-based settings for mass screening of children for acute malnutrition and referral for those below set cut-offs (< 12·5 cm for moderate acute malnutrition and < 11·5 cm for severe acute malnutrition). Given the ease with which MUAC can be measured and that it does not require calibrated equipment, it may be more practical than WAZ or WLZ even if it has slightly worse performance. In 2023, the updated WHO guidelines included low MUAC (< 11·0 cm) as a criterion for identifying nutritionally at-risk infants aged 6 weeks to 6 months but did not include MUAC for identification of at-risk neonates. Previous evidence has suggested a range of MUAC values between 11·5 and 10·5 cm to be optimal for identifying wasting in infants under 6 months of age^(3)^. In line with these results, the present analysis identified a cut-off of 10·5 cm in neonates aged 8–27 d, although MUAC did not perform as well as WAZ or WLZ for predicting mortality. However, the practicalities of MUAC measurement may outweigh slightly reduced performance. Further research evaluating MUAC as a criterion for identifying nutritionally at-risk neonates that includes the smallest infants is warranted^(17)^.

Although low birth-weight infants had higher mortality than normal birth-weight infants, birth weight did not perform as well for identifying children at risk of mortality as other neonatal anthropometric measures^(18)^. Low birth weight can be a result of preterm birth or restricted intra-uterine growth, and its aetiology is diverse. The present study was unable to measure gestational age due to lack of ultrasound or last menstrual period data. As babies had to be at least 8 d of age to be eligible for the study, those who were born and died in their first week of life were not included in this cohort. Results therefore may not be generalisable to neonates in their first week of life and may underestimate the relationship between low birth weight and mortality. Low birth weight in the cohort was associated with poor growth outcomes at 6 months and was more strongly associated with stunting and underweight than with wasting, in line with previous evidence from sub-Saharan Africa^(19)^.

This analysis must be considered in the context of several limitations. Data arose from a randomised controlled trial designed to evaluate the efficacy of neonatal azithromycin for the prevention of infant mortality. Inclusion criteria prioritised safety of research participants, specifically with regard to risk of infantile hypertrophic pyloric stenosis. As some studies have suggested that smaller infants may be at increased risk of infantile hypertrophic pyloric stenosis^(20,21)^, infants weighing less than 2500 g at the time of enrolment (age 8–27 d) were not eligible for the trial. As a result, this cohort was likely better nourished than the general population of infants in Burkina Faso, and some enrolment groups, such as low WAZ, were relatively small. However, this large cohort with enrolment from across Burkina Faso provides valuable data regarding the utility of neonatal anthropometric measurements for identifying at-risk infants. Future studies with population representative samples should be conducted to confirm these results, and results should be interpreted with the limitation that the most vulnerable neonates were not included in this study. Data were collected as part of a large randomised controlled trial, and results may not be generalisable to non-trial settings. Trials often represent best-case scenarios, as they typically have resources that are not available under real-life conditions. As previously noted, equipment used for anthropometric measurement may be poorly calibrated or have low precision for measuring small infants^(16)^. Analysis of anthropometric indicators using routinely collected programmatic data may be valuable data to understand the performance of these indicators under real-world conditions. Analyses of birth weight should be interpreted with caution. Infants who died before being screened for the trial may have been more likely to be low birth weight, thus biasing results. Birth weight measures were not standardised, as they were extracted from government-issued health cards and not measured by trained study staff. As a result, there may be more measurement error in the birth weight measures than in anthropometric measures collected by the trial. Birth weight measurements were missing for some children, either because they were not born in a health facility and thus did not have their birth weight measured, or because their health card was missing. If these infants were more likely to be low birth weight and more likely to die or have worse growth compared with those born in a facility, there could be bias introduced via missing birth weight measurements. However, the prevalence of missing birth weight was relatively low (2·4 %), and thus unlikely to introduce substantial bias.

Overall, the results of this study suggest that, in line with previous evidence, neonatal WAZ was the strongest predictor of mortality by 6 months of age. Low WAZ was also highly predictive of other anthropometric deficits at 6 months of age, including stunting and wasting. However, given the potential limitations of measuring WAZ, especially outside of trial settings, additional studies evaluating MUAC in neonates, including the smallest infants, should be considered.

## Supporting information

Bountogo et al. supplementary material Bountogo et al. supplementary material

## Supplementary material

For supplementary material accompanying this paper visit https://doi.org/10.1017/S1368980024000880
