# Supplementary material for: Neonatal anthropometric indicators of infant growth and mortality in Burkina Faso
Source: Public Health Nutr. 2024 Apr 19;27(1):e123. doi: 10.1017/S1368980024000880 (PMC11091928; doi:10.1017/S1368980024000880)
Supplement: Bountogo et al. supplementary material [file S1368980024000880sup001.docx]

**Supplemental Table S1.** Associations between baseline anthropometric measures and mortality at 6 months among infants who were low birthweight (< 2500 g) or not low birthweight (≥ 2500 g).

|  | Low birthweight (<2500 g) | | | Not low birthweight (≥2500 g) | | |
| --- | --- | --- | --- | --- | --- | --- |
|  | N | Number Died (%) | Odds Ratio  (95% CI) | N | Number Died (%) | Odds Ratio^1^  (95% CI) |
| **WAZ** |  |  |  |  |  |  |
| ≥ - 2 | 934 | 7 (0.72%) | 1.00 | 18,017 | 61 (0.32%) | 1.00 |
| < -2 and ≥ -3 | 622 | 6 (0.93%) | 1.29 (0.40 to 4.09) | 743 | 11 (1.42%) | 4.75 (2.32 to 8.90) |
| < -3 | 98 | 3 (2.88%) | 4.40 (0.65 to 31.20) | 47 | 1 (2.0%) | 9.60 (0.51 to 54.91) |
| AUC (continuous) |  |  | 0.59 (0.44 to 0.74) |  |  | 0.67 (0.60 to 0.73) |
| **WLZ** |  |  |  |  |  |  |
| ≥ - 2 | 1,187 | 6 (0.48%) | 1.00 | 16,477 | 56 (0.33%) | 1.00 |
| < -2 and ≥ -3 | 309 | 5 (1.59%) | 3.31 (0.95 to 11.07) | 1,692 | 9 (0.52%) | 1.57 (0.72 to 3.02) |
| < -3 | 134 | 4 (2.90%) | 6.27 (1.58 to 22.40) | 611 | 8 (1.25%) | 3.88 (1.70 to 7.71) |
| AUC (continuous) |  |  | 0.66 (0.51 to 0.82) |  |  | 0.62 (0.58 to 0.69) |
| **LAZ** |  |  |  |  |  |  |
| ≥ - 2 | 1,079 | 12 (1.08%) | 1.00 | 17,838 | 67 (0.36%) | 1.00 |
| < -2 and ≥ -3 | 444 | 2 (0.43%) | 0.34 (0.05 to 1.28) | 857 | 4 (0.44%) | 1.28 (0.39 to 3.14) |
| < -3 | 131 | 2 (1.44%) | 0.94 (0.13 to 4.08) | 112 | 2 (1.74%) | 5.07 (0.82 to 16.82) |
| AUC (continuous) |  |  | 0.45 (0.28 to 0.62) |  |  | 0.56 (0.49 to 0.62) |
| **MUAC** |  |  |  |  |  |  |
| ≥ 11.5 cm | 164 | 2 (1.18%) | 1.00 | 6,112 | 14 (0.22%) | 1.00 |
| < 11.5 to ≥ 10.5 cm | 444 | 2 (0.43%) | 0.40 (0.05 to 3.27) | 6,571 | 22 (0.32%) | 1.48 (0.76 to 2.97) |
| < 10.5 to ≥ 9.5 cm | 644 | 10 (1.51%) | 1.40 (0.36 to 9.24) | 5,011 | 26 (0.50%) | 2.32 (1.22 to 4.61) |
| < 9.5 cm | 397 | 2 (0.49%) | 0.44 (0.05 to 3.72) | 1,039 | 11 (1.03%) | 4.78 (2.10 to 10.61) |
| AUC (continuous) |  |  | 0.47 (0.34 to 0.60) |  |  | 0.61 (0.54 to 0.67) |

Abbreviations: AUC, area under a receiver operating characteristic curve; WAZ, weight-for-age Z-score; WLZ, weight-for-length Z-score; LAZ, length-for-age Z-score; MUAC, mid-upper arm circumference; ^1^Adjusted for child’s age in days at enrollment and sex
